# Phase I study of camrelizumab in patients with advanced solid tumors

**DOI:** 10.1038/s41392-022-01213-6

**Published:** 2023-02-01

**Authors:** Yuxiang Ma, Jiaxin Cao, Yang Zhang, Qianwen Liu, Wenfeng Fang, Yunpeng Yang, Yuanyuan Zhao, Qing Yang, Hongyun Zhao, Li Zhang

**Affiliations:** 1 Department of Clinical Research, Sun Yat-sen University Cancer Center, State Key Laboratory of Oncology in South China, Collaborative Innovation Center for Cancer Medicine, Guangdong Key Laboratory of Nasopharyngeal Carcinoma Diagnosis and Therapy, Guangzhou, People’s Republic of China; 2 Department of Medical Oncology, Sun Yat-sen University Cancer Center, State Key Laboratory of Oncology in South China, Collaborative Innovation Center for Cancer Medicine, Guangdong Key Laboratory of Nasopharyngeal Carcinoma Diagnosis and Therapy, Guangzhou, People’s Republic of China; 3 Jiangsu Hengrui Medicine Co. Ltd, Lianyungang, People’s Republic of China

**Keywords:** Drug development, Immunotherapy

**Dear Editor**,

Camrelizumab (SHR-1210) is a humanized monoclonal antibody (mAb) that binds to programmed cell death protein 1 (PD-1).^1^ Since May 2019, camrelizumab has been successfully approved for the therapy of patients with various malignancies.^2^ However, only a few studies have focused on the pharmacokinetics (PK), pharmacodynamics (PD), and immunogenicity of camrelizumab and its potential impact on clinical outcomes in patients with advanced malignant solid tumors. In mAb-treated patients, generated anti-drug antibodies (ADAs) which bind to therapeutic proteins may impact the PK or safety of the therapeutic drug and eventually lead to a loss of efficacy. A previous study reported that receptor occupancy (RO) of mAbs is a critical predictor of its efficacy. RO analysis helps to determine the level of biological effect of treatment, so as to prevent adverse reactions.^3^ The purpose of this study was to evaluate the PK parameters of camrelizumab in a Chinese population. The PD-1 RO rate in peripheral blood CD3+T cells, the incidence of ADA, the association of RO and ADA, safety and efficacy were all assessed. PK, PD, and immunogenicity analysis were performed in samples obtained from patients enrolled in a dose-escalation (a standard 3 + 3 design) and expansion stage phase I trial performed at Sun Yat-sen University Cancer Center (NCT02721589). Patients were treated with camrelizumab at doses of 1 mg/kg, 3 mg/kg, 10 mg/kg, or a 200 mg flat-dose every 2 weeks.

Forty-nine patients were incorporated in the PK evaluation (Supplementary Fig. 1). Baseline characteristics and baseline weight are summarized in Supplementary Tables 1 and 2. Baseline characteristics (including sex) did not differ significantly between the four cohorts. The main PK parameters (the area under the concentration time curve from zero to the last time of quantifiable concentration (AUC_0-last_), the area under the concentration time curve from zero to time infinity (AUC_0-inf_), the maximum concentration (C_max_), the time to maximum concentration (T_max_), the minimum or maximum concentration at a steady state (C_ss,min_, C_ss,max_), elimination half-life (t_1/2_), clearance (CL), volume of distribution (V_d_), mean residence time (MRT), and accumulation ratio (Rac)) are summarized in Supplementary Tables 3 (single-dose administration) and 4 (multiple-dose administration). The C_max_, AUC, t_1/2_, CL, and V_d_ between the 200 mg flat-dose and the 3 mg/kg dose were similar. The plasma concentration-time curve of the 200 mg flat-dose was similar with the 3 mg/kg dose (Supplementary Fig. 2).

The RO rate 5 minutes after a single dosing was about 80–88%. After multiple dosing of camrelizumab for about 3–5 treatment cycles, the RO rate essentially stabilized. The RO rate of each dose group remained at a high level before discontinuation, and then decreased significantly over time (Supplementary Fig. 3 and Supplementary Table 5). There was no statistical difference in progression-free survival (PFS) (1.87 vs 1.95 vs 4.1 vs 3 months, *p* = 0.1592) and overall survival (OS) (14.83 vs 13.47 vs 18.53 vs 7.43 months, *p* = 0.5568, date not shown) between the four dose groups (1 mg/kg, 3 mg/kg, 200 mg flat-dose, and 10 mg/kg). The optimum cut-off values of the highest RO for PFS obtained using X-tile was 98.1% (Fig. 1a–c). Patients’ highest RO that was below 98.1% had a significantly shorter PFS compared with those with a highest RO that was above 98.1% (median PFS, 1.867 vs 5.350 months, *p* < 0.0001; Fig. 1d). The OS in patients with a higher RO was longer than that of patients with a lower RO (median OS, 18.00 vs 11.53 months, *p* = 0.0114; Fig. 1e). PFS differed significantly between patients with a mean RO above 72.45% and those with a mean RO below 72.45% (median PFS: 3.8667 vs 1.7167 months *p* = 0.0006, Supplementary Fig. 4a). Patients’ lowest RO that was above 13.6%, and patients’ lowest RO that was below 13.6% were statistically different (median PFS: 2.85 vs 1.8667 months *p* = 0.0388, Supplementary Fig. 4c). However, the appropriate cut-off values of the mean and lowest RO of each patient for OS obtained by X-tile were not statistically significant (Supplementary Fig. 4b–d). Adverse events (AEs) also did not affect the efficacy of camrelizumab (Supplementary Fig. 4e, f).

**Fig. 1:**
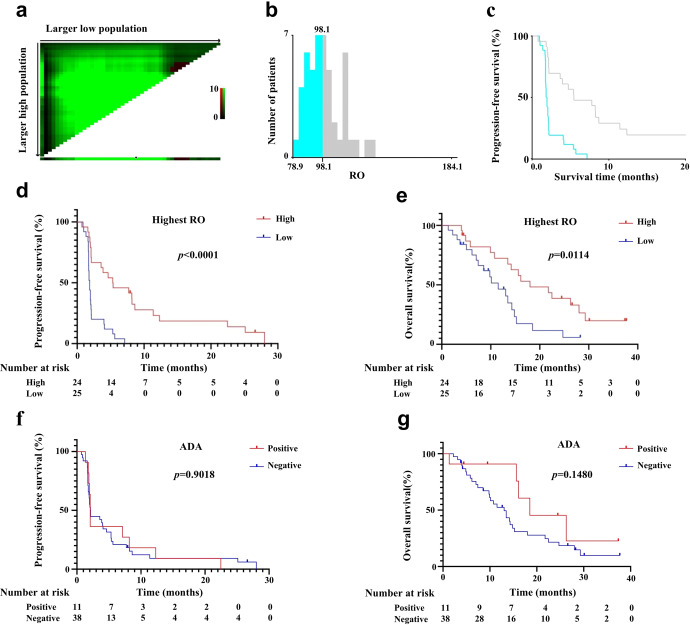
The survival curves of highest RO stratified by the cutoff values calculated by X-tile. **a** The X-tile plots in the left panels show the χ2 log-rank values, which represent all possible divisions of the lipid levels into low or high. The *X*-axis represents the low population, and the *Y*-axis the high population. **b** The optimal cut-off values for the highest RO of each patient for PFS obtained using X-tile software was 98.1%. **c** The optimal cut-off values are shown in the Kaplan–Meier plots. **d** PFS differed significantly between patients with a highest RO that was above 98.1%, and those with a highest RO that was below 98.1% (median PFS: 5.350 vs 1.867 months *p* < 0.0001). **e** OS differed significantly between patients’ highest RO that was above 98.1% and patients’ highest RO that was below 98.1% (median OS: 18.00 vs 11.53 months *p* = 0.0114). **f** PFS was not significantly different for ADA positive and ADA-negative patients (2.086 vs 2.037 months *p* = 0.9018). **g** OS was not significantly different for ADA positive and ADA-negative patients (18.53 vs 13.03 months *p* = 0.1480)

ADA incidences of camrelizumab were evaluated in 49 patients; 20.4% of patients had at least one ADA-positive sample (Supplementary Table 6). No significant impact of ADA on PFS and OS were found among the four dose groups (Fig. 1f, g). All 49 patients (100%) had at least one treatment-related AE, the adverse events involved are summarized in Supplementary Table 7. Spearman’s rank correlation estimated that there was no association between ADA or RO with AE grades 1–3 (Supplementary Table 8). Taken together, the ADA were not significantly associated with PFS, OS, or AEs.

To our knowledge, the study is the first and largest cohort to report systemic PK analysis for camrelizumab, in revealing the predictive values of RO and ADA in advanced solid tumor patients. Based on the PK results, the dosing groups of 200 mg and 3 mg/kg every 2 weeks exhibited similar drug exposure in this study. Both weight-based dosing regimen and flat dosing regimen were suitable for camrelizumab, which is consistent with a previous study.^4^ Flat dosing is more convenient, safer, and more compliant than weight-based dosing and may be more appropriate in clinical settings.

RO is an important observational measure in clinical trials of immunotherapy drug development. The PD-1 receptor is the basic theory for the formation of the tumor inhibition effect of camrelizumab. A high PD-1 RO provides a consistent blockade of PD-1, and may promote better antineoplastic effect.^5^ The results of the RO analysis showed that the high affinity of camrelizumab to the PD-1 receptor was consistent with the results of in vitro experiments, and patients with higher RO levels were associated with better PFS and OS. The efficacy of mAbs on PD-1 receptors is mediated by intra-tumoral RO. However, it is difficult to obtain continuous intra-tumor RO in clinical practice. Analyzing intra-tumor RO results from a single puncture is also limited. Our study evaluated the impact of continuous peripheral blood RO on the efficacy of camrelizumab, although it may not fully reflect the intra-tumor and differentiated non-activated T cells in RO, it still provides a better understanding of the therapeutic efficacy of camrelizumab and RO.

In this study, 20.4% of patients were ADA positive. The presence of ADAs may be associated with hypersensitivity, infusion reactions, and loss of efficacy. But no significant effects of positive-ADA on safety or efficacy were observed. The incidence of ADA is closely related to the sensitivity and specificity of the detection method and is affected by many factors, including the analysis method, the sample handling method, the sample collection time, the drug combination, and other underlying diseases of the patient. Due to this study’s small sample size, the impact of ADA on the safety and efficacy needs to be studied in larger samples.

The RO level may be a potential biomarker for indicating the clinical response of immunotherapy. Developing biomarkers that promote the clinical outcome of immunotherapy remains a hot area of research, in which it is crucial to evaluate and maximize the benefit versus risk ratio of drugs in treatment choice. Further studies, including exposure-response studies are needed to better inform clinical dosing strategies.

## Supplementary information

Supplementary Materials

## Data Availability

The data generated and analyzed is available from the corresponding author upon reasonable request. The authenticity of this article has been validated with key raw data uploaded onto the Research Data Deposit (RDD) public platform (https://www.researchdata.org.cn). The approval RDD number is RDDA2022135024.
