# Supplementary material for: Phase I study of camrelizumab in patients with advanced solid tumors
Source: Signal Transduct Target Ther. 2023 Feb 1;8:47. doi: 10.1038/s41392-022-01213-6 (PMC9892498; doi:10.1038/s41392-022-01213-6)
Supplement: Supplementary file 1 — Supplementary Materials [file 41392_2022_1213_MOESM1_ESM.docx]

Supplementary Materials for

Phase I Study of Camrelizumab in Patients with Advanced Solid Tumors

Yuxiang Ma^1^*, Jiaxin Cao^1^*, Yang Zhang^1^*, Qianwen Liu^1^*, Wenfeng Fang^2^, Yunpeng Yang^2^, Yuanyuan Zhao^2^, Qing Yang^3^, Hongyun Zhao^1#^, Li Zhang^2#^

* These authors contributed equally to this work.

**^#^** Correspondence to: Li Zhang [zhangli](mailto:zhangli6@mail.sysu.edu.cn)@sysucc.org.cn or

Hongyun Zhao [zhaohy@sysucc.org.cn](mailto:zhaohy@sysucc.org.cn)

**This PDF file includes:**

Materials and Methods

Figures S1 to S4

Tables S1 to S8

**Materials and methods**

Sample collection

Serum samples were collected at prespecified time points. In the first cycle, a total of 9 blood samples for PK were collected from each patient, including 0.5 hours before initiation-of-infusion (IOI), and 5 minutes after, 2, 6, 24, and 48 hours after, and on days 8, 15, and 22 after IOI. Seven blood samples for RO were collected from each patient, including 0.5 h before IOI, 5 minutes after, and on days 2, 3, 8, 15, and 22 after IOI. Four ADA blood samples were collected from each patient, 0.5 h before IOI, and on days 8, 15, and 22 after IOI. From the second cycle, blood samples for PK and RO were collected 0.5 hours before IOI and 5 minutes after IOI in the third cycle of each 3-cycle. ADA blood samples were collected 0.5 hours before IOI in the third cycle of each 3-cycle, after disease progression or treatment discontinuation, and once a month after discontinuation for 3 months.

PK evaluation

The serum concentrations of camrelizumab were measured using Enzyme-Linked Immunosorbent Assay (ELISA) by Lapcorp Pharmaceutical (Shanghai) Co., LTD (previous name Covance). The concentrations of the corrected standard curve ranged from 157 to 10000 ng/mL. The calculated PK parameters included the area under the concentration time curve from zero to the last time of quantifiable concentration (AUC_0-last_), the area under the concentration time curve from zero to time infinity (AUC_0-inf_), the maximum concentration (C_max_), the time to maximum concentration (T_max_), the minimum or maximum concentration at a steady state (C_ss,min_, C_ss,max_), elimination half-life (t_1/2_), clearance (CL), volume of distribution (V_d_), mean residence time (MRT), and accumulation ratio (Rac). The non-compartment analysis (NCA) method was used to calculate the PK parameters.

PD evaluation

Flow cytometry was used to detect the PD-1 receptor occupancy (RO) rate on peripheral blood T cells. The method was developed by WuXi Apptec. (Suzhou) Co., LTD. (non-GLP) and transferred to WuXi Apptec. (Shanghai) Co., LTD. (non-GLP). As is well-known, it is difficult to comprehensively detect PD-1 occupancy in all immune cells. The current strategy is to detect a subgroup of immune cells which constitutes a large proportion that is stable, repeatable, and that is a suitable drug surrogate. CD3+ cells are dominant immune cells in peripheral blood and PD-1 expression is in ~20% of T-cells,^1^ which provides us with a suitable indicator reflecting the dynamics of SHR-1210 in vivo. Similarly, RO in a Nivolumab Phase I study also used CD3+ cells as RO target cells.^2^ At present, the RO of PD-1 in peripheral blood CD3+ cells is the most reflective of the overall situation.

The T cell receptor occupancy rate was calculated by RO=A/B×%, where A is the blood sample *in vitro* on the CD3 T cells without adding the test substance and B is the percentage of the blood sample on CD3 T cells when the test substance is added to the blood sample *in vitro*. PD-1 receptor occupancy is defined as the percentage of the PD-1 receptor occupied by camrelizumab in peripheral blood T cells.

ADA evaluation

A validated bridging electrochemiluminescence (ECL) immunoassay was used for the detection of anti-camrelizumab antibodies in human serum by Lapcorp Pharmaceutical (Shanghai) Co., LTD (previous name Covance). ADA bioanalysis of camrelizumab was carried out using the standard 3-tiered assay approach that consisted of screening (Tier 1), confirmation (Tier 2), and antibody titer assessment (Tier 3).

The immunogenicity status of patients was derived on the basis of the longitudinal ADA status of the post-baseline samples. Patients with one or more post-baseline positive ADA samples were classified as ADA-positive. Patients whose post-baseline ADA samples were all negative were classified as ADA-negative. ADA positive patients were divided into three categories: transiently positive, consistently positive, and other positive. The ADA status of patients was categorized according to the following definitions. Transient ADA positivity is defined as negative at baseline, and having only one post-baseline ADA-positive sample before the last sample, and is not consistently ADA-positive. Consistent ADA positivity consists of two categories (baseline negative): (1) at least two post-baseline positive samples in which the interval between the first and the last ADA-positive samples is ≥16 weeks; and (2) the last post-baseline sample is positive or the last post-baseline sample is negative, and the second to last sample is positive before the last sample is ADA-negative. Other ADA positivity indicates negative at baseline, having at least two post-baseline ADA-positive samples, that are not consistently ADA positive, and the last sample is ADA-negative.

Clinical outcome assessment

In the camrelizumab monotherapy trial, the primary endpoint was to determine the safety and tolerability of camrelizumab for common solid tumors and the recommended phase 2 dose. Secondary endpoints included pharmacokinetic parameters of camrelizumab via intravenous injection at a fixed dose versus a weight based calculated dose; evaluation of the PD-1 receptor occupancy on the surface of the peripheral blood T cells after treatment; measurement of anti-camrelizumab antibody concentrations in serum in patients after treatment; and preliminary evaluation of the antitumor activity of camrelizumab for solid tumors of high incidence in China.

Progression-free survival (PFS) was defined as the period from the first dose to RECIST-assessed progression or death from any cause, whichever occurred first. Overall survival (OS) was defined as the period from enrollment to death from any cause. The data cut-off date for the present analysis was November 17, 2019. AEs were monitored from the signing of informed consent until 90 days after the last dose and graded according to the Common Terminology Criteria for Adverse Events (CTCAE) v4.03.

Statistical analyses

PK and PD parameters of camrelizumab were calculated by the non-compartmental method using WinNonlin 7.0 software. The relationship of dose proportionality response was evaluated using the power model: log(y) = β0 + β1*log(dose), with y representing the single-dose PK parameter (AUC_0-last_, AUC_0-inf_, C_max_), and β0 representing the intercept term. The 90% confidence interval (CI) of the slope β1 was estimated using a mixed-effects model with the treatment group as a fixed effect. The statistical analyses were conducted using SPSS software, version 22 (IBM, Armonk, NY, USA) and GraphPad Prism 8 (GraphPad Software, La Jolla, CA, USA). The association between RO, ADA, and AEs were estimated using Spearman’s rank correlation. We used X-tile software to evaluate the cutoff value of the lowest, mean and highest RO for all patients as the optimal value for predicting PFS. X-Tile operational software (Yale University, New Haven, CT, USA) provided precise statistical estimates for dividing cases according the "low" or "high" expression of specific biomarkers.^3^ Survival was evaluated using the Kaplan-Meier method. A threshold of P < 0.05 for all statistics was considered a significant difference.

**
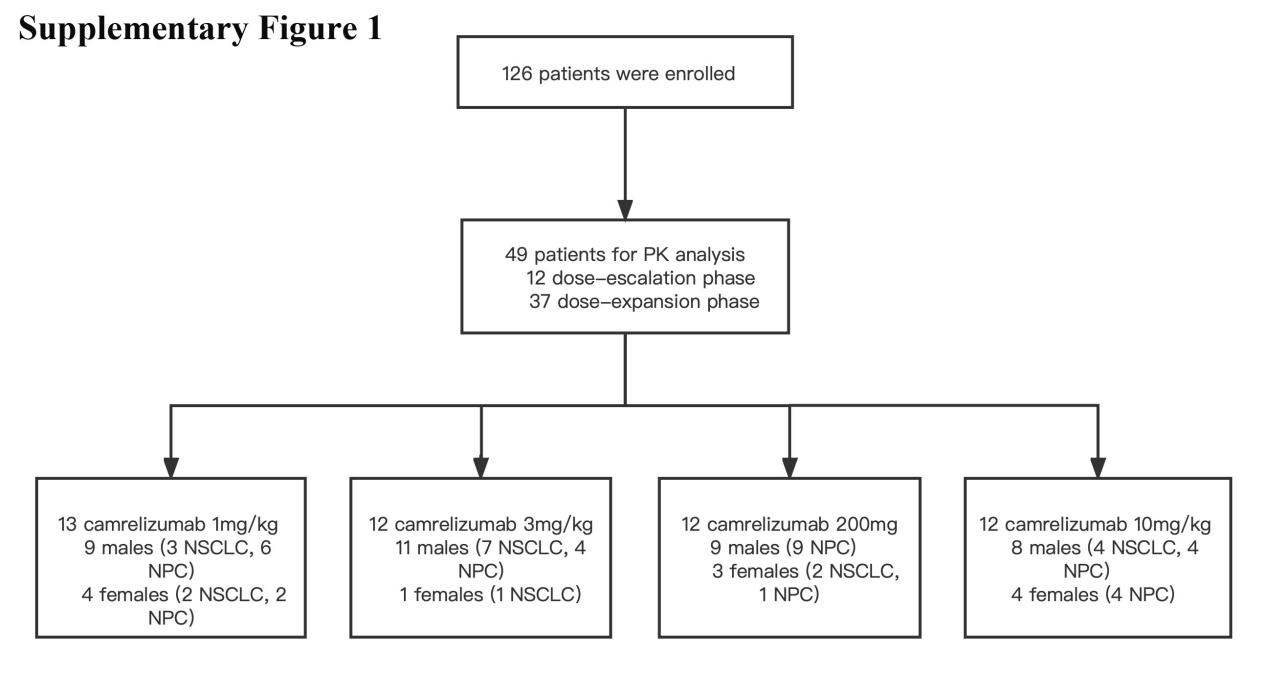
**

**Supplementary Figure 1**. Study profile. NSCLC, non-small-cell lung cancer; NPC, nasopharyngeal carcinoma; PK, pharmacokinetics.

**
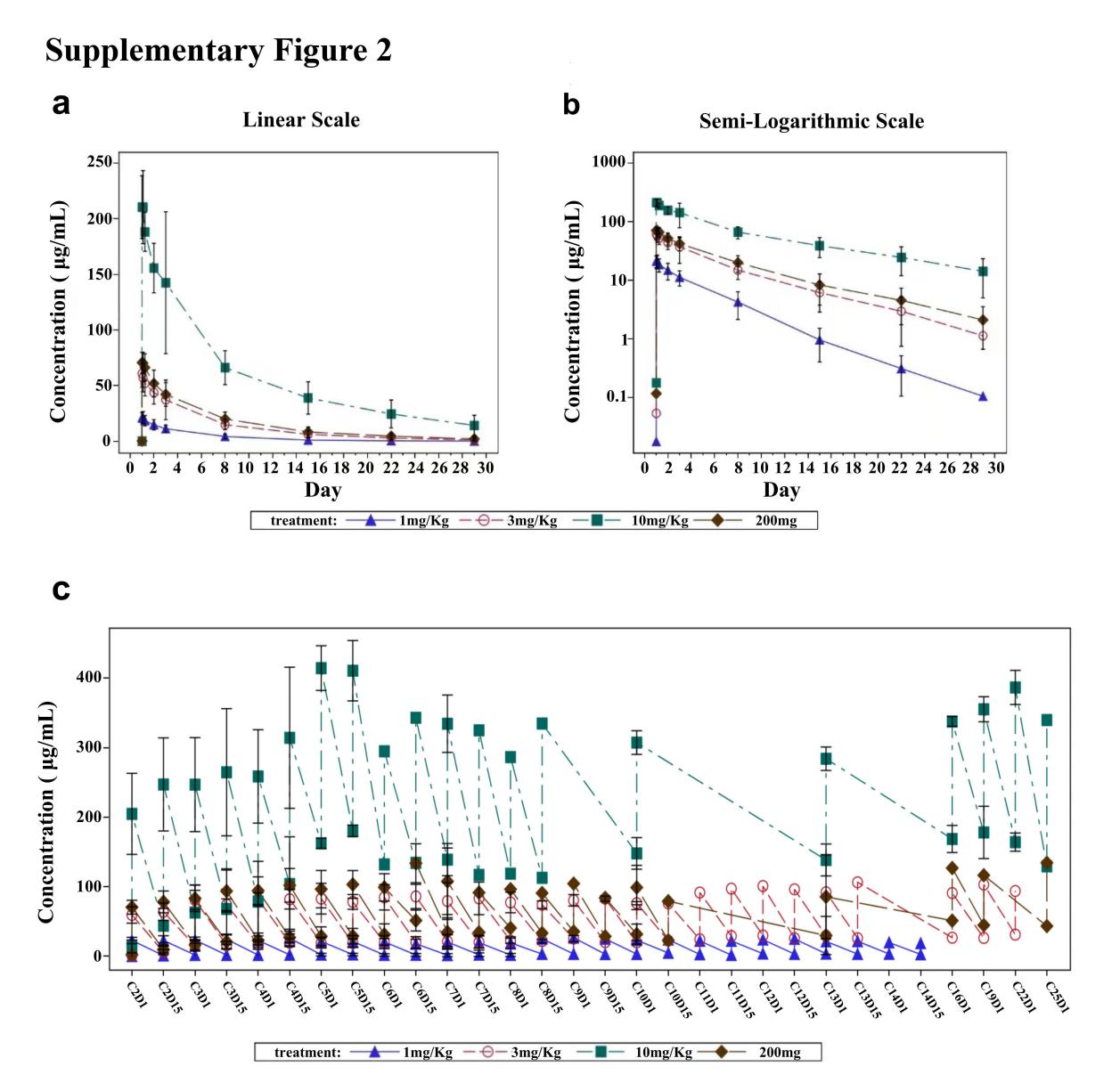
**

**Supplementary Figure 2**. Mean concentration-time curve of SHR-1210 in serum of patients in each dose group after administration. (a) Mean concentration-time curve in each dose group after single administration with the form of linear scale. (b) Mean concentration-time curve in each dose group after single administration with the form of semi-logarithmic scale. (c) Mean concentration-time curve in each dose group after multiple administration.

**
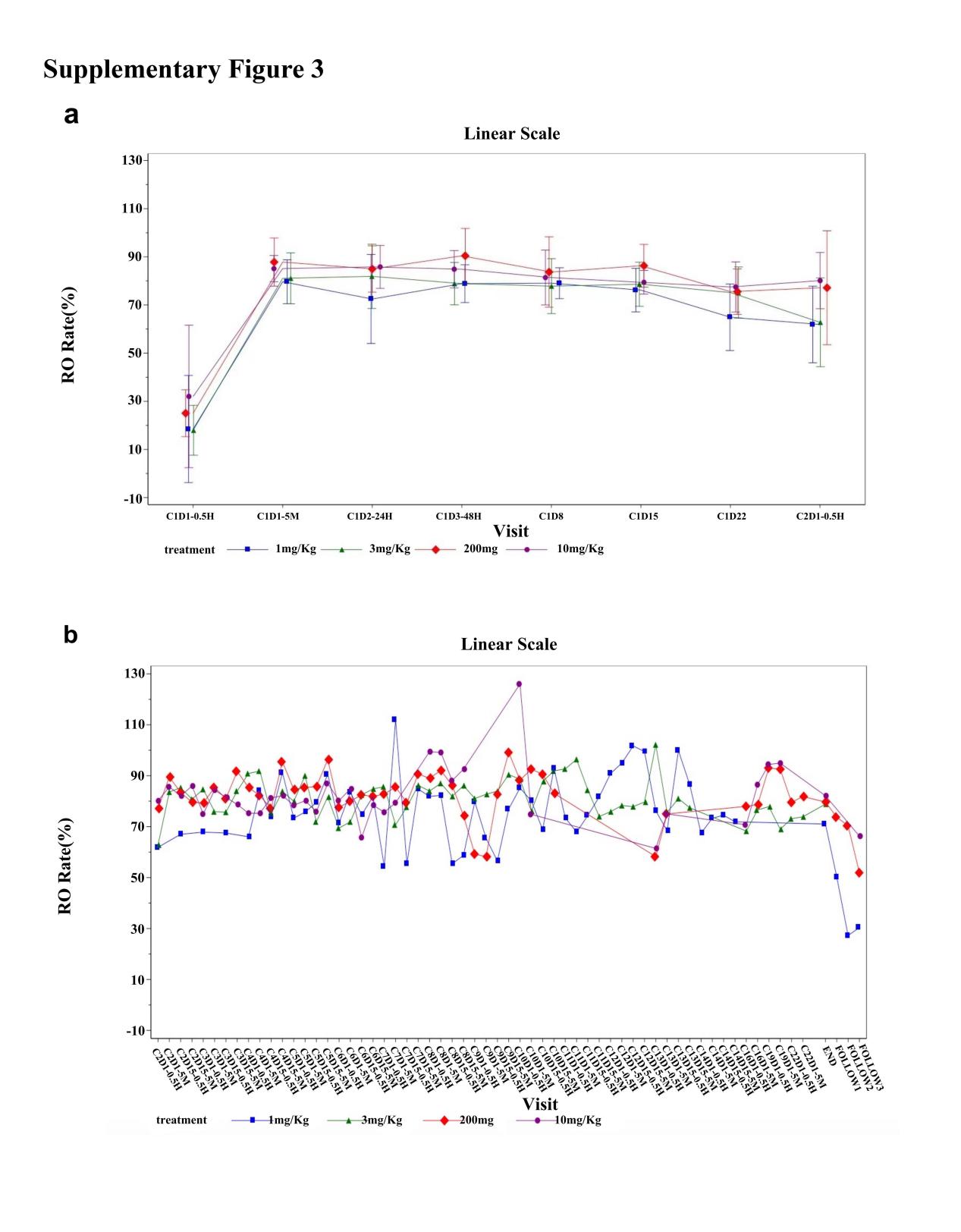
**

**Supplementary Figure S3**. The average RO rate of camrelizumab in each dose group after administration. (a) Average RO rate in each dose group after single administration. (b) Average RO rate in each dose group after multiple administration.

**
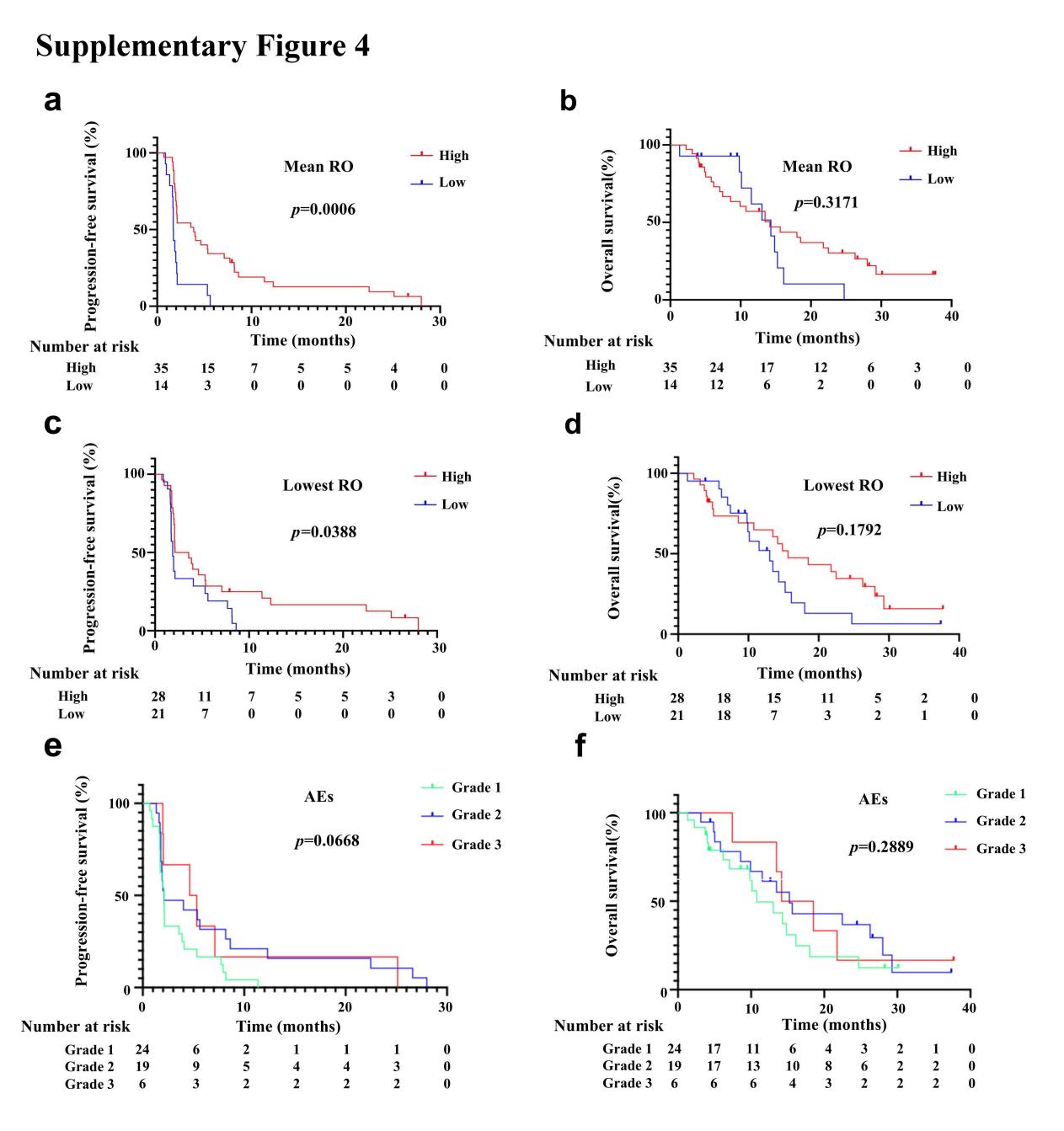
**

**Supplementary Figure S4**. The survival curves of the mean and lowest RO stratified by the cutoff values calculated by X-tile. (a) PFS differed significantly between patients with a mean RO above 72.45 % and those with a mean RO below 72.45 % (median PFS: 3.8667 vs 1.7167 months P =0.006). (b) OS was the similar between patients with a mean RO above 72.45% and those with a mean RO below 72.45% (median OS: 14.1667 vs 14.3 months P = 0.3171). (c) PFS differed significantly between patients’ lowest RO that was above 13.6 % and patient’s lowest RO that was below 13.6 % (median PFS: 2.85 vs 1.8667 months P =0.0388). (d) OS was not significantly different between patients’ lowest RO that was above 13.6 % and patient’s lowest RO that was below 13.6 % (15.6333 vs 13.0333 months P = 0.1792). (e) PFS was not significantly different between patients with Grade 1-3 AEs (median PFS: 2.07 vs 2.07, 4.98 months P =0.0668). (f) OS was not significantly different between patients with Grade 1-3 AEs (10.77 vs 15.23, 16.35 months P = 0.2889).

**Table S1. Characteristics of all patients.**

| **Characteristics** | **1 mg/kg** | **3 mg/kg** | **200 mg** | **10 mg/kg** | ***p*** |
| --- | --- | --- | --- | --- | --- |
|  | **N (%)** | **N (%)** | **N (%)** | **N (%)** |  |
| **Age (years)** |  |  |  |  | 0.9252 |
| Median (range) | 48 (33–61) | 51 (34–66) | 47 (37–60) | 37 (23–69) |  |
| ＜60 | 11 (84.6) | 10 (83.3) | 11 (91.7) | 10 (83.3) |  |
| ≥60 | 2 (15.4) | 2 (16.7) | 1 (8.3) | 2 (16.7) |  |
| **Gender** |  |  |  |  | 0.4789 |
| Male | 9 (69.2) | 11 (91.7) | 9 (75) | 8 (66.7) |  |
| Female | 4 (30.8) | 1 (8.3) | 3 (25) | 4 (33.3) |  |
| **ECOG** |  |  |  |  | 0.8873 |
| 0 | 6 (46.2) | 5 (41.7) | 4 (33.3) | 4 (33.3) |  |
| 1 | 7 (53.8) | 7 (58.3) | 8 (66.7) | 8 (66.7) |  |
| **Smoking status** |  |  |  |  | 0.1452 |
| Non-smokers | 9 (69.2) | 4 (33.3) | 9 (75) | 8 (66.7) |  |
| Smokers | 4 (30.8) | 8 (66.7) | 3 (25) | 4 (33.3) |  |
| **Distant metastasis** |  |  |  |  | 0.8244 |
| Liver | 8 (61.5) | 6 (50) | 7 (58.3) | 7 (58.3) |  |
| Lung | 9 (69.2) | 3 (25) | 5 (41.7) | 9 (75) |  |
| Bone | 6 (46.2) | 6 (50) | 7 (58.3) | 5 (41.7) |  |
| Distant lymph nodes | 10 (76.9) | 10 (83.3) | 11 (91.7) | 10 (83.3) |  |
| Pleura  Others | 1 (7.7) | 5 (41.7) | 3 (25) | 1 (8.4) |  |
| Others | 1 (7.7) | 2 (16.6) | 1 (8.4) | 2 (16.6) |  |
| **Previous chemotherapy** |  |  |  |  | 0.7580 |
| Induction | 5 (38.5) | 3 (25) | 6 (50) | 9 (75) |  |
| Concurrent | 6 (46.2) | 3 (25) | 6 (50) | 9 (75) |  |
| palliative | 13 (100) | 12 (100) | 12 (100) | 12 (100) |  |
| **Previous lines of therapy** |  |  |  |  | 0.1225 |
| 1 | 7 (53.8) | 4 (33.3) | 1 (8.4) | 4 (33.3) |  |
| 2 | 1 (7.7) | 3 (25) | 3 (25) | 3 (25) |  |
| 3 | 1 (7.7) | 1 (8.4) | 6 (50) | 4 (33.3) |  |
| 4 or more | 4 (30.8) | 4 (33.3) | 2 (16.6) | 1 (8.4) |  |
| **Previous Radiotherapy** |  |  |  |  | 0.3369 |
| No | 4 (30.8) | 7 (58.3) | 4 (33.3) | 3 (25) |  |
| Yes | 9 (69.2) | 5 (41.7) | 8 (66.7) | 9 (75) |  |
| **Tumor type** |  |  |  |  | 0.0876 |
| NSCLC | 5 (38.5) | 8 (66.7) | 2 (16.7) | 4 (33.3) |  |
| NPC | 8 (61.5) | 4 (33.3) | 10 (83.3) | 8 (66.7) |  |

**Abbreviations:** ECOG, Eastern Cooperative Oncology Group; NSCLC, non–small cell lung cancer; NPC, nasopharyngeal carcinoma.

**Table S2 Statistical summary of body weight (kg) in each dose group.**

| **Group** | **N** | **Mean (kg)** | **SD (kg)** | **Median (kg)** | **Min (kg)** | **Max (kg)** |
| --- | --- | --- | --- | --- | --- | --- |
| **1 mg/kg** | 13 | 54.662 | 8.5159 | 53.800 | 36.80 | 66.70 |
| **3 mg/kg** | 12 | 57.808 | 7.5070 | 59.250 | 46.00 | 68.10 |
| **200 mg** | 12 | 57.550 | 7.3747 | 57.350 | 42.80 | 72.10 |
| **10 mg/kg** | 12 | 54.892 | 7.2496 | 54.900 | 44.60 | 65.80 |

**Table S3. Summary of PK parameters of SHR-1210 in serum of each dose group after single dose administration.**

| **PK parameters(unit)** | | **1 mg/kg**  **(N=13)** | **3 mg/kg**  **(N=12)** | **200 mg**  **(N=12)** | **10 mg/kg**  **(N=12)** |
| --- | --- | --- | --- | --- | --- |
| C_max_ (μg/mL) | Mean±SD | 21.867±4.9726 | 61.564±12.5731 | 73.572±11.4634 | 228.846±43.6472 |
|  | Geomean  (%CV Geomean) | 21.291  (25.38) | 60.483  (19.53) | 72.715  (16.34) | 225.400  (17.97) |
| T_max_ (hour) | Median  (Min, Max) | 2.50 (0.58,2.60) | 0.58 (0.58, 48.50) | 2.50 (0.55, 6.50) | 2.50 (0.58, 48.50) |
| AUC_0-last_ (day*μg/mL) | Mean±SD | 85.8447±31.6420 | 324.3784±114.2742 | 410.8221±140.5004 | 1524.7101±327.6381 |
|  | Geomean  (%CV Geomean) | 80.2468  (40.95) | 307.8571  (34.23) | 387.7627  (37.61) | 1491.5562  (22.43) |
| AUC_0-inf_  (day*μg/mL) | Mean±SD | 87.3314±31.9196 | 335.7255±125.0404 | 431.0858±156.6520 | 1745.3814±489.0085 |
|  | Geomean  (%CV Geomean) | 81.7451  (40.45) | 316.7783  (36.17) | 404.5321  (39.40) | 1680.9513  (29.64) |
| t _½_ (Day) | Mean±SD | 3.59±0.75 | 5.46±1.64 | 5.77±1.98 | 9.17±3.16 |
| CL (L/day) | Mean±SD | 0.6972±0.2488 | 0.5853±0.2326 | 0.5289±0.2089 | 0.3379±0.1040 |
|  | Median | 0.6728 | 0.5222 | 0.4653 | 0.3121 |
| V_d_ (L) | Mean±SD | 3.5507±1.5388 | 4.2848±1.2915 | 3.9837±1.1006 | 4.1103±0.7123 |
|  | Median | 3.3316 | 4.0308 | 3.7444 | 3.8387 |
| MRT (day) | Mean±SD | 4.96±0.87 | 7.19±2.16 | 7.77±2.34 | 12.12±4.36 |

**Abbreviations:** PK, pharmacokinetic; AUC_0-last_, area under the concentration-time curve (0 to last measurable concentration); AUC_0-inf_, area under the concentration-time curve (0 to infinity); C_max_, maximum concentration; C_ss,min_, minimum concentration at steady state; C_ss,max_, maximum concentration at steady state; T_max_, time to maximum concentration; t_1/2_, elimination half-life; CL, clearance; V_d_, volumn of distribution; MRT, mean residence time; SD, standard deviation.

**Table S4. Summary of the PK parameters of SHR-1210 in serum of Cycle 4 Day 15 after multiple dose administration in each dose group.**

| **PK parameters (unit)** | | **1 mg/kg (N=13)** | **3 mg/kg**  **(N=12)** | **200 mg**  **(N=12)** | **10 mg/kg**  **(N=12)** |
| --- | --- | --- | --- | --- | --- |
|  |  | **n=6** | **n=5** | **n=6** | **n=3** |
| C_ss,min_  (μg/mL) | Mean±SD | 2.113±1.4043 | 19.570±5.8458 | 27.138±11.7119 | 103.990±67.8732 |
|  | Geomean  (%CV Geomean) | 1.595 (114.50) | 18.735 (35.90) | 24.858 (50.52) | 81.813 (122.21) |
| C_ss,max_  (μg/mL) | Mean±SD | 25.952±6.2261 | 82.024±14.1217 | 102.067±24.1879 | 314.227±101.5219 |
|  | Geomean  (%CV Geomean) | 25.271 (26.36) | 81.120 (16.47) | 99.483 (25.83) | 302.033 (36.69) |
| Rac | Mean±SD | 1.106±0.2618 | 1.247±0.1103 | 1.351±0.2262 | 1.277±0.5884 |
|  | Geomean  (%CV Geomean) | 1.080 (24.02) | 1.243 (9.12) | 1.337 (15.68) | 1.161 (62.21) |

**Abbreviations:** PK, pharmacokinetic; C_ss,min_, minimum concentration at steady state; C_ss,max_, maximum concentration at steady state; Rac, accumulation ratio; CV, coefficient of variation; SD, standard deviation. N= Number of subjects in the analysis population; n= Number of subjects that meet a particular category.

**Table S5. Average RO rate of Camrelizumab.**

| **Time** | **Mean** | **1 mg/kg** | **3 mg/kg** | **200 mg** | **10 mg/kg** |
| --- | --- | --- | --- | --- | --- |
|  | **(coefficient of variation%, n)** | **(N=13)** | **(N=12)** | **(N=12)** | **(N=12)** |
| C1D1  (5 minutes after administration) | Mean | 80% | 81% | 88% | 85% |
|  | (%CV, n） | (11.44, 13) | (13.06, 12) | (11.37, 11) | (6.37, 12) |
| C1D2  (24 hours after administration) | Mean | 72% | 82% | 85% | 86% |
|  | (%CV, n） | (25.52, 13) | (16.29, 12) | (11.35, 11) | (10.40, 12) |
| C1D15  (15 days after administration) | Mean | 76% | 79% | 86% | 79% |
|  | (%CV, n） | (11.86, 13) | (11.65, 12) | (10.26, 12) | (6.20, 11) |
| C1D22  (22 days after administration) | Mean | 65% | 75% | 76% | 77% |
|  | (%CV, n） | (21.29, 12) | (14.13, 12) | (12.52, 11) | (13.45, 12) |
| C2D1  (0.5 hours after administration) | Mean | 62% | 63% | 77% | 80% |
|  | (%CV, n） | （25.70, 10） | (29.38, 12) | (30.67, 10) | (14.60, 12) |
| C4D1  (0.5 hours after administration) | Mean | 66% | 91% | 85% | 75% |
|  | (%CV, n） | （11.23, 7） | (27.38, 5) | (16.90, 7) | (10.74, 6) |
| C4D15  (0.5 hours after administration) | Mean | 74% | 75% | 77% | 81% |
|  | (%CV, n） | （13.98, 6） | (13.59, 5) | (17.16, 6) | (7.84, 3) |
| C4D15  (0.5 hours after administration) | Mean | 91% | 84% | 95% | 82% |
|  | (%CV, n） | （ -, 1） | (8.45, 3) | (7.97, 4) | (4.37, 3) |
| END  (Out of group) | Mean | 71% | 79% | 80% | 82% |
|  | (%CV, n） | （16.41, 10） | (15.95, 8) | (10.65, 6) | (17.02, 5) |

**Abbreviations:** RO, receptor occupancy; CV, coefficient of variation.

**Table S6. Summary of ADA incidence.**

| **ADA positive classification** | | **1 mg/kg  (N=13)** | **3 mg/kg  (N=12)** | **200 mg  (N=12)** | **10 mg/kg  (N=12)** | **Total  (N=49)** |
| --- | --- | --- | --- | --- | --- | --- |
| Baseline ADA positive | n（%） | 2 (15.4%) | 0 | 1 (8.3%) | 0 | 3 (6.1%) |
|  | 95% CI | (1.9, 45.4) | (0.0, 26.5) | (0.2, 38.5) | (0.0, 26.5) | (1.3, 16.9) |
| Baseline ADA negative and post-baseline ADA positive | n（%） | 2 (15.4%) | 2 (16.7%) | 3 (25.0%) | 0 | 7 (14.3%) |
|  | 95% CI | (1.9, 45.4) | (2.1, 48.4) | (5.5, 57.2) | (0.0, 26.5) | (5.9, 27.2) |
| Transiently ADA positive | n（%) | 2 (15.4%) | 1 (8.3%) | 1 (8.3%) | 0 | 4 (8.2%) |
|  | 95% CI | (1.9, 45.4) | (0.2, 38.5) | (0.2, 38.5) | (0.0, 26.5) | (2.3, 19.6) |
| Consistently ADA positive | n（%） | 0 | 1 (8.3%) | 1 (8.3%) | 0 | 2 (4.1%) |
|  | 95% CI | (0.0, 24.7) | (0.2, 38.5) | (0.2, 38.5) | (0.0, 26.5) | (0.5, 14.0) |
| Other ADA positive | n（%） | 0 | 0 | 1 (8.3%) | 0 | 1 (2.0%) |
|  | 95% CI | (0.0, 24.7) | (0.0, 26.5) | (0.2, 38.5) | (0.0, 26.5) | (0.1, 10.9) |

**Abbreviations:** ADA, anti-drug antibody; CI, confidence interval.

Table S7. Treatment-related adverse events.

| **Adverse events** | **Patients (%)** |
| --- | --- |
| Anemia | 4 (8.2) |
| Anorexia | 4 (8.2) |
| Nausea | 3 (6.1) |
| Albuminuria | 3 (6.1) |
| Peripheral neuropathy | 1 (2.1) |
| Rash | 17 (34.7) |
| Oedema | 3 (6.1) |
| Hyponatremia | 1 (2.1) |
| Hypochloremia | 1 (2.1) |
| Vomit | 3 (6.1) |
| Constipation | 3 (6.1) |
| Fatigue | 20 (40.8) |
| Hypoalbuminemia | 1 (2.1) |
| Dizziness | 2 (4.1) |
| Pruritus | 11 (22.4) |
| Fever | 7 (14.3) |
| Hypercreatinine | 1 (2.1) |
| Diarrhea | 6 (12.2) |
| Reactive capillary hemangiomas | 30 (61.2) |
| Hypokalemia | 1 (2.1) |
| Total bilirubin elevation | 1 (2.1) |
| Thyroid stimulating hormone concentration decrease | 6 (12.2) |

Table S8. Relationships between ADA, RO and AE.

|  | **ADA** | | | **RO** | | |
| --- | --- | --- | --- | --- | --- | --- |
| **Characteristic** | **Negative**  (n=38, 77.6%) | **Positive**  (n=38, 22.4%) | ***p* Value** | **High**  (n=28, 57.1%) | **Low**  (n=21, 42.9%) | ***p* Value** |
| The grade of AE | n (%) |  |  |  |  |  |
| 1 (n=24, 49%) | 19 (38.8) | 5 (10.2) | 0.917 | 11 (22.4) | 13 (26.5) | 0.082 |
| 2 (n=19, 38.8%) | 14 (28.6) | 5 (10.2) | **（1.157-5.5）** | 12 (24.5) | 7 (14.4) |  |
| 3 (n=6, 12.2%) | 5 (10.2) | 1(2) |  | 5 (10.2) | 1 (2) |  |

**Abbreviations:** ADA, anti-drug antibody; RO, receptor occupancy; AEs, adverse events.

**References**

1. Davidson TB, Lee A, Hsu M, et al. Expression of PD-1 by T Cells in Malignant Glioma Patients Reflects Exhaustion and Activation. *Clin Cancer Res.* **25**, 1913-1922 (2019).

2. Hotchkiss RS, Colston E, Yende S, et al. Immune checkpoint inhibition in sepsis: a Phase 1b randomized study to evaluate the safety, tolerability, pharmacokinetics, and pharmacodynamics of nivolumab. *Intensive Care Med.* **45**, 1360-13719 (2019).

3. Camp RL, Dolled-Filhart M, Rimm DL. X-tile: a new bio-informatics tool for biomarker assessment and outcome-based cut-point optimization. *Clin Cancer Res.* **10**, 7252-9 (2004).
